# FTY-720 induces apoptosis in neuroblastoma via multiple signaling pathways

**DOI:** 10.18632/oncotarget.22452

**Published:** 2017-11-06

**Authors:** Ingo Lange, Italo Espinoza-Fuenzalida, Mourad Wagdy Ali, Laura Espana Serrano, Dana-Lynn T. Koomoa

**Affiliations:** ^1^ University of Hawaii at Hilo, The Daniel K. Inouye College of Pharmacy, Hilo, HI 96720, USA

**Keywords:** neuroblastoma, calcium, FTY720, TRPM7, channel

## Abstract

Neuroblastoma (NB) is the most common extra-cranial pediatric solid tumor. High-risk NB is difficult to treat due to the lack of response to current therapies and aggressive disease progression. Despite novel drugs, alternative treatments and multi-modal treatments, finding an effective treatment strategy for these patients continues to be a major challenge. The current study focuses on examining the effects of FTY-720 or fingolimod, a drug that is FDA-approved for refractory multiple sclerosis, in NB. The results showed that FTY-720 regulates multiple pathways that result in various effects on calcium signaling, ion channel activation and cell survival/death pathways. FTY-720 rapidly inhibits TRPM7 channel activity, and inhibited TRPM7 kinase activity, modulates calcium signaling, induces a loss of mitochondrial membrane potential and opening of the mitochondrial permeability transition pore, and ultimately leads to cell death. Interestingly, the data also showed that low concentrations of FTY-720 sensitized drug-resistant NB cells to antineoplastic drugs. These results suggest that FTY-720 may be an attractive alternative for the treatment of NB.

## INTRODUCTION

Neuroblastoma (NB) is the most common extra-cranial pediatric solid tumor. NB is highly heterogeneous, with clinical outcomes that range from spontaneous regression to the development of multi-drug resistance. High-risk NB (∼40–50% of patients) signifies poor prognosis, and is difficult to treat due to the lack of response to current therapies and aggressive disease progression [1–3]. Numerous studies have examined the effects of novel drugs, alternative treatments and multi-modal treatments for patients with high-risk and relapsed NB. However, finding an effective treatment strategy for these patients continues to be a major challenge due to the aggressive phenotypes of these tumors and a myriad of complex mechanisms that promote resistance to treatments and recurrence [3–6]. The current study focuses on examining the effects of FTY-720 or fingolimod, a drug that is FDA-approved for refractory multiple sclerosis [7–11], in NB. Previous studies have shown that FTY-720 inhibits NB tumor growth by inhibiting sphingosine kinase 2 [12], and FTY-720 inhibits TRPM7 in HEK-293 cells stably expressing TRPM7 [13]. This study examined the effects of FTY-720 on TRPM7 in NB cells.

The results showed that FTY-720 treatment resulted in multiple effects in NB cells including alterations in calcium signaling, ion channel activation, and NB cell death. FTY-720 rapidly inhibits TRPM7 channel activity and inhibited TRPM7 kinase activity in NB cells. FTY-720 also modulates intracellular free calcium levels and mitochondrial calcium, in a dose-dependent and time-dependent manner. Interestingly, higher concentrations of FTY-720 induced a loss of mitochondrial membrane potential, and the opening of the mitochondrial permeability transition pore. This suggests that FTY-720 may cause mitochondrial damage. Interestingly, at low concentrations of FTY-720 NB cells may induce autophagy to remove damaged mitochondria and promote NB cell survival [14, 15]. However, at higher concentrations of FTY-720, the damage to mitochondria may be extensive and autophagy may not be sufficient to maintain NB cell viability.

Interestingly, the data also showed that low concentrations of FTY-720 sensitized drug-resistant NB cells to doxorubicin and other antineoplastic drugs. This effect makes FTY-720 an attractive alternative for the treatment of NB patients, as it is effective on multiple NB cell lines, including the drug-resistant cell line SK-N-Be(2)c.

In summary, FTY-720 induces numerous changes in NB cells, including loss of TRPM7 channel and kinase activities, changes in calcium signaling, loss of mitochondrial membrane potential and plasma membrane potential, that subsequently induces apoptosis.

## RESULTS

### FTY-720 decreases cell viability

**Figure 1:**
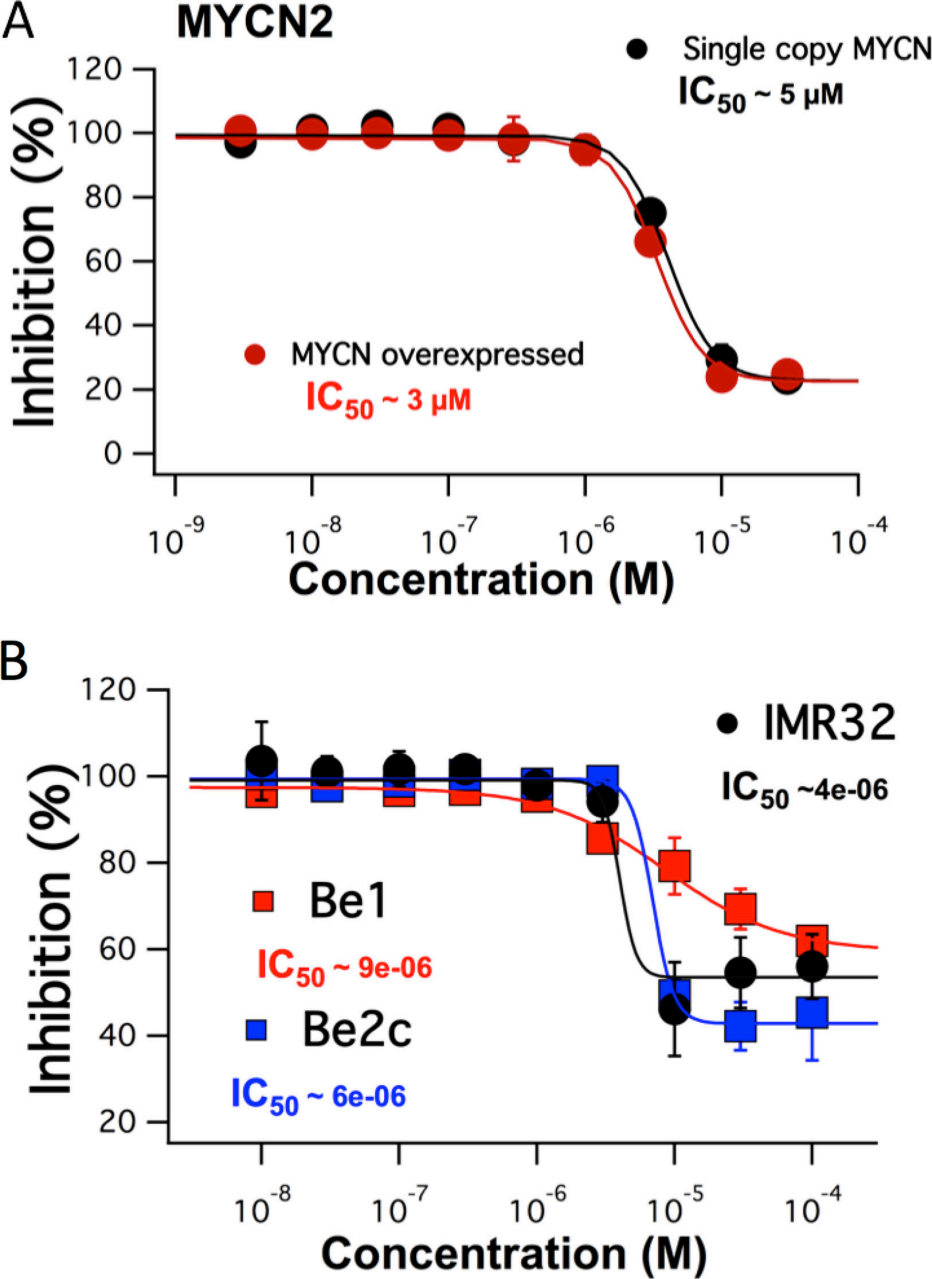
The dose dependent effects of FTY-720 on MYCN2 cells with and without MYCN overexpression (**A**) and in IMR32, SK-N-Be1 and SK-N-Be(2)c (**B**) obtained using MTS assays. All experiments were performed *n* = 3.

In order to determine the anti-cancer effects of FTY-720 in SHEP-1 NB cells, cells were treated with FTY-720, doxorubicin as a positive control, or left untreated for 48 hours. The effects of the various treatments were then assessed using the Sulforhodamine B (SRB), MTS assay and Trypan blue staining. The results showed that FTY-720 significantly decreased overall NB cell numbers, compared to untreated control (data not shown). The MTS assay was used to determine the overall dose dependent effects of FTY-720 on NB cell viability and proliferation. The half maximal inhibitory concentration (IC_50_) was then determined for FTY-720. Figure 1 shows that FTY-720 had a dose-dependent effect on NB cells. The IC_50_ of SHEP-1 NB cells with and without MYCN over-expression were 3 and 5 μM FTY-720, respectively. Under same conditions a SRB assay was performed yielding similar half-maximal concentrations for FTY-720 of 2 μM and 4 μM with and without overexpression of MYCN respectively (Supplementary Figure 1A). A subsequent Trypan blue staining at 10 and 20 μM FTY-720 compared to control indicated impact on cell viability (data not shown). In order to test whether FTY-720 impairs the viability of different NB cell lines with MYCN-amplified status, dose-response IC_50_ values were established for IMR32, SK-N-Be1 and SK-N-Be(2)c cell lines. The MTS assay generated half maximal values at 4 μm for IMR32 and 6 μM and 9 μM for SK-N-Be1 and SK-N-Be(2)c (Figure 1B). The three cell lines displayed impaired cell viability indicated by Trypan blue staining at 10 μM and 20 μM FTY-720 compared to untreated cells (data not shown). SK-N-Be1 is a MYCN-amplified cell line isolated from the same patient as SK-N-Be(2)c but before the development of multi-drug resistance. Western blot analysis comparing expression of TRPM7 among the 2 cell lines pairs was performed in order to explain the difference in IC_50_ values. TRPM7 was significantly higher expressed in drug-resistant SK-N-Be(2)c compared to SK-N-Be1 indicating a stronger effect of FTY-720 and shift of IC_50_ values for these cell line pairs (Supplementary Figure 2).

Similar results were observed when the dose dependent effects were MYCN gene amplified NB cells isolated after patient relapse, SK-N-Be(2)c, were either pre-treated with FTY-720 or left untreated for 12 hours, and the dose dependent effects of doxorubicin was determined using the SRB assay. The results suggest that FTY-720 sensitized the cells to doxorubicin, and shifted the IC_50_ from 200nM without FTY-720 pretreatment to 4 nM with FTY-720 pretreatment (Supplementary Figure 1B). The data suggests that FTY-720 may decrease NB cell viability, and may sensitize drug-resistant NB to antineoplastic chemotherapeutic drugs.

### FTY-720 induces apoptosis and autophagy

**Figure 2:**
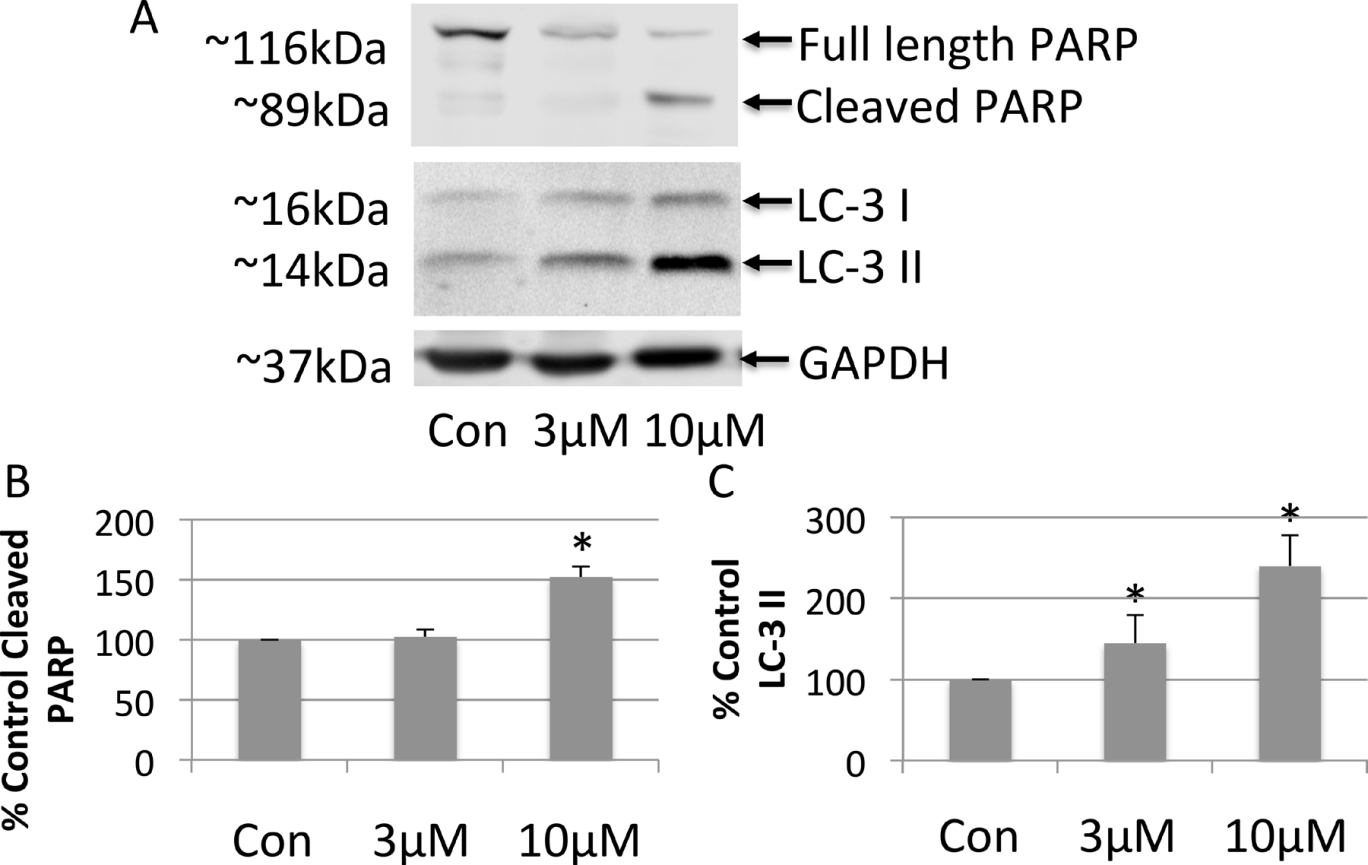
Western blot analysis of whole cell lysates prepared from neuroblastoma cells treated with 3 μM or 10 μM FTY-720 (**A**). The bands were quantified and the mean and standard deviation are presented in panels (**B** and **C)**. All experiments were performed *n* = 3.

To investigate the mechanism whereby FTY-720 exerts its anticancer effects in SHEP-1 NB, cell lysates were analyzed by western blot. Whole cell lysates were prepared from cells after 24 hour treatment with doxorubicin, 3 μM FTY-720, 10 μM FTY-720, and from untreated control cells. The proteins were separated by SDS-page and transferred onto nitrocellulose membranes. The effects of the different treatments were analyzed by western blot in order to determine changes in the expression of proliferation, apoptosis and autophagy markers. FTY-720 did not alter cell proliferation markers (data not shown). Interestingly, 10 μM FTY-720 induced PARP cleavage (Figure 2A and 2B) suggesting that FTY-720 induced apoptosis. Similar results were obtained with 20 μM FTY-720 (data not shown). This effect was not observed in cells treated with 3 μM FTY-720. Interestingly, 3 μM and 10 μM FTY-720 also induced the processing of microtubule-associated protein IA/B-light chain 3, LC3 and increased LC3-II, a marker for autophagy (Figure 2A and 2C). Similar results were obtained with 20 μM FTY-720 (data not shown). These results suggest that low concentrations of FTY-720 induce autophagy, while higher concentrations induce both autophagy and apoptosis.

### FTY-720 inhibits TRPM7 channel and kinase activities

**Figure 3:**
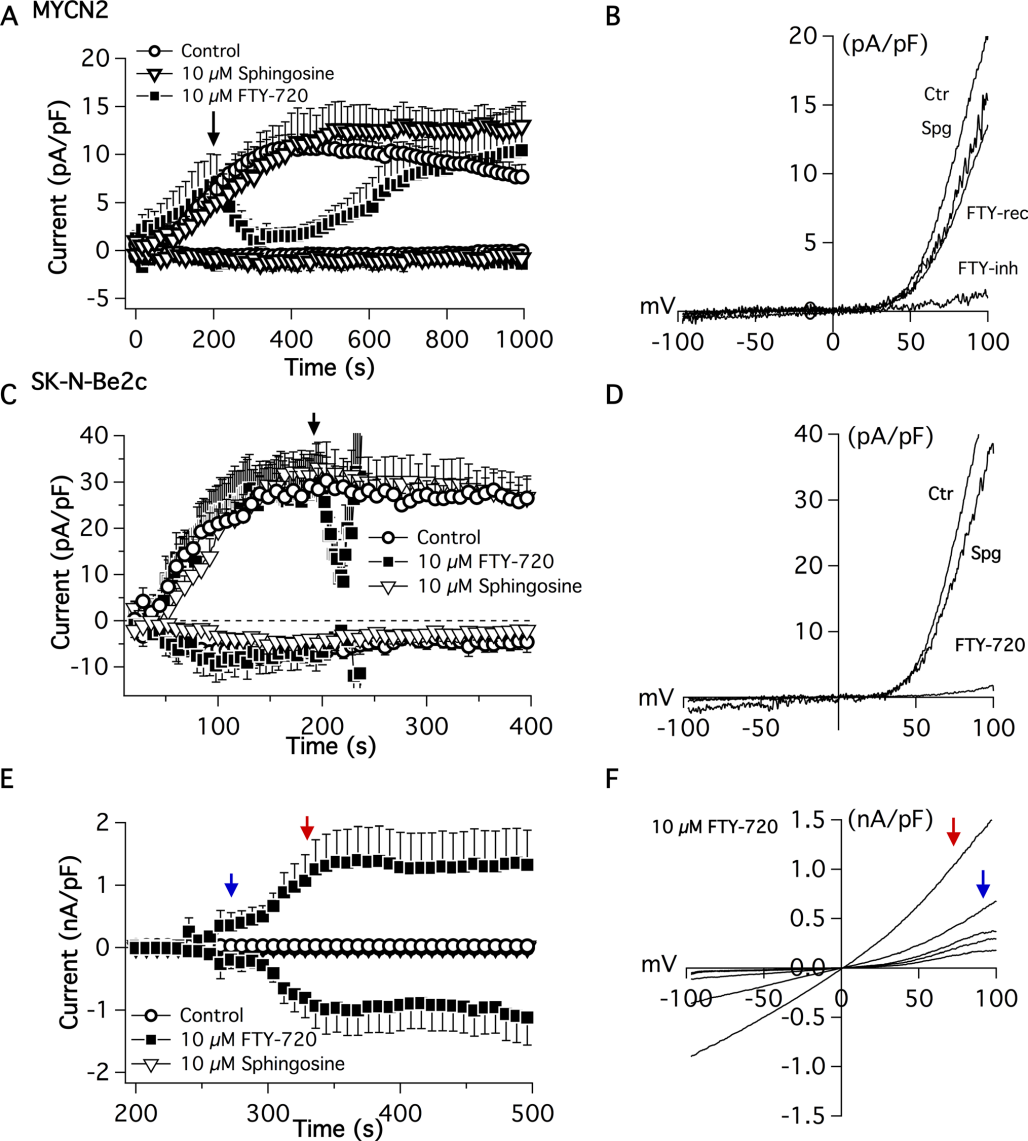
Electrophysiological patch-clamp measurements were recorded in MYCN2 and SK-N-Be(2c) cells treated with FTY-720 or vehicle control FTY-720 inhibits TRPM7 currents (*n* = 8–12).

To determine the effects of FTY-720 on TRPM7 channel activity, whole cell electrophysiological patch clamp measurements were recorded in SHEP-1 NB cells. TRPM7 current density was determined at +80 mV on NB cells (Figure 3A and 3B). External application of 10 μM FTY-720 on NB cells decreased the current density rapidly and was restored upon cessation of FTY-720 application (Figure 3A and 3B). The characteristic TRPM7 current/voltage relationship (IV curve) was observed, displaying a large outwardly rectifying current, and a very small inward current. Similar results were obtained with external application of 10 μM FTY-720 on SK-N-Be(2)c cells (Figure 3C and 3D). Interestingly, 3 μM FTY-720 showed moderate or no inhibition of TRPM7 (data not shown), and 20 μM FTY-720 induced the activation of another channel with an IV curve that resembles that of a TRP-like channel (Figure 3E and 3F). FTY-720 is an analogue of sphingosine; therefore, in order to determine if the effects of FTY-720 are due to sphingosine signaling, sphingosine was used as an added control. The results show that sphingosine did not have an effect on TRPM7 channel activity (Figure 3A–3CA, 3E and 3F). The data suggest that FTY-720 inhibits TRPM7 channel activity at low concentrations. However, at higher (20 μM) concentrations, FTY-720 activates another current

**Figure 4:**
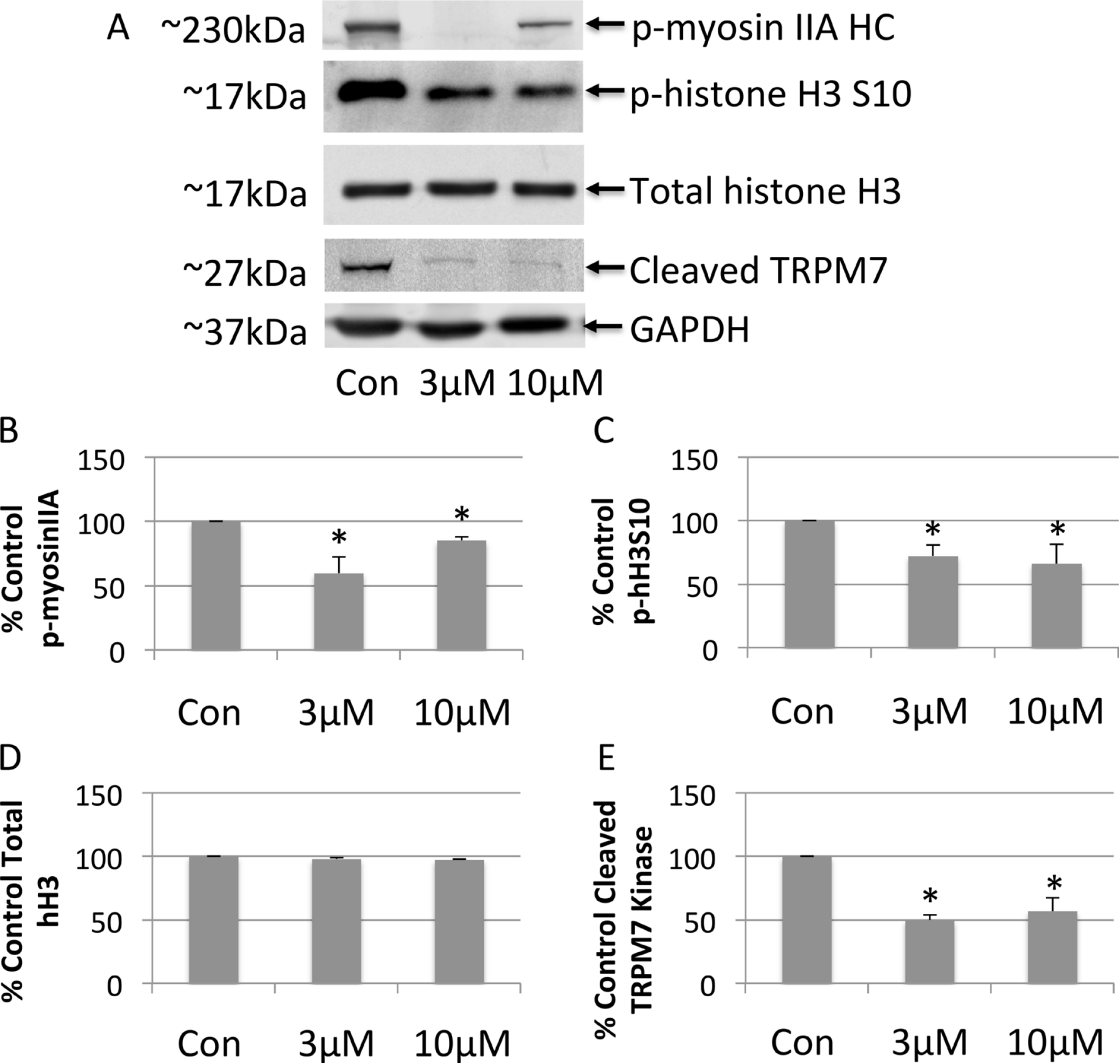
Western blot analysis of whole cell lysates prepared from neuroblastoma cells treated with 3 μM or 10 μM FTY-720 (**A**). The bands were quantified and the mean and standard deviation are presented in panels (**B**–**E)**. All experiments were performed *n* = 3.

Next, the effects of FTY-720 on TRPM7 kinase activity was determined using western blot. Whole cell lysates were prepared from NB cells treated with 3 μM FTY-720, 10 μM FTY-720, and from control untreated NB cells. The proteins were separated by SDS-page and transferred onto nitrocellulose membranes. The effects of the different treatments were analyzed by western blot in order to determine changes in the phosphorylation of TRPM7 kinase targets, myosin IIA heavy chain and histone H3, as well as the cleavage of the TRPM7 kinase domain. The results show that 3 μM FTY-720 decreased the phosphorylation of myosin IIA and histone H3 by 40% and 28%, respectively (Figure 4A–4D). 3 μM FTY-720 also decreased the cleavage of the TRPM7 kinase by ∼50% (Figure 4A–4D). 10 μM FTY-720 reduced the phosphorylation of myosin IIA and histone H3 by 15% and 34%, respectively (Figure 4A–4D). 10 μM FTY-720 also decreased the cleavage of the TRPM7 kinase by ∼44% (Figure 4A–4D). Similar results were obtained with 20 μM FTY-720 (data not shown). To further analyze myosin IIA and histone H3 phosphorylation by TRPM7, NB cells were treated with scrambled siRNA, TRPM7 specific siRNA, or mock transfected (lipofectamine only). Whole cell lysates were prepared from these cells. The proteins were separated by SDS-page and transferred onto nitrocellulose membranes. The effects of the scrambled and TRPM7 siRNA were analyzed by western blot in order to determine changes in the phosphorylation of TRPM7 kinase targets, myosin IIA heavy chain and histone H3. The results show that TRPM7 siRNA significantly decreased TRPM7 protein expression (Supplementary Figure 2), compared to scrambled siRNA. TRPM7 siRNA also significantly decreased the phosphorylation of myosin IIA and histone H3, respectively (Supplementary Figure 2). The data suggest that FTY-720 reduces the TRPM7 kinase activity.

### FTY-720 modulates calcium signaling and alters membrane integrity

**Figure 5:**
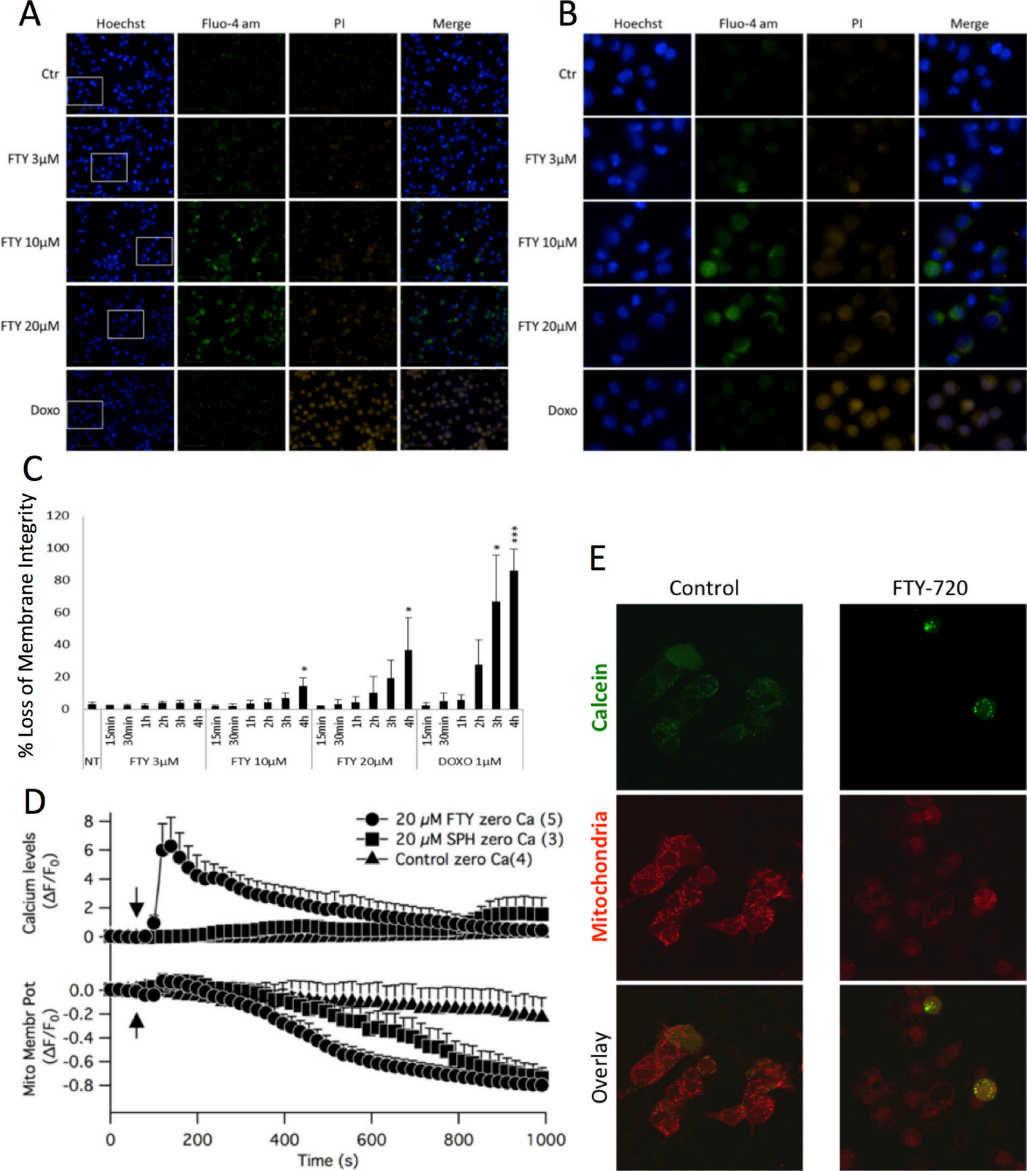
Epifluorescent images were acquired of neuroblastoma cells treated with 3 μM or 10 μM FTY-720, and labeled with fluo-4, Hoechst and propidium iodide (**A** and **B**). The fluorescence intensities were quantified and the mean and standard deviation are presented in panels (**C**). Mitochondrial membrane potential measurements were obtained by staining cells with TMRE (**D**). Opening of the mitochondrial permeability transition pore was examined by staining cells with Calcein AM and MitoTracker (**E**). All experiments were performed *n* = 3.

Next, the effect of FTY-720 on intracellular free calcium was determined using the calcium indicator dye, Fluo-4. The relative fluorescence was measured in Fluo-4-AM loaded cells treated with FTY-720 or untreated control cells. Hoechst 33342 was used to label the nucleus of all cells, and propidium iodide was used to examine plasma membrane integrity. Ionomycin and doxorubicin were used as positive controls for calcium mobilization and induction of cell death, respectively. Immunofluorescent images were obtained using the Perkin Elmer High Throughput Operetta Imaging System. The results showed that 10 and 20 μM FTY-720 increased calcium signaling in SHEP-1 NB cells within 4 hours of treatment, as shown by the increase in Fluo-4 intensity (Figure 5A and 5B). This was not observed at 3 μM FTY-720, which is the IC_50_ concentration of FTY-720 (shown in Figure 1). In addition, the plasma membrane integrity was compromised within 4 hours of 10 and 20 μM FTY-720 treatment, as shown by the increase in propidium iodide positive cells (Figure 5A and 5B). This effect was not observed at 3 μM FTY-720. The fluorescence intensities of propidium iodide was quantified and plotted in Figure 5C. The results showed that 10 and 20 μM FTY-720 significantly reduced plasma membrane integrity (Figure 5C). The results suggest that at 10 and 20 μM FTY-720, there is an alteration in calcium signaling and loss of plasma membrane integrity that may cause a reduction in NB cell viability.

To determine the effects of FTY-720 on mitochondrial membrane potential, SHEP-1 NB cells were loaded with Fluo-4 and TMRE. Time lapsed imaging was performed using a laser scanning confocal microscope. The results show that 20 μM FTY-720 induced a rapid increase in intracellular free calcium levels (Figure 5D). In addition, 20 μM FTY-720 also induced a loss of mitochondrial membrane potential, decreasing TMRE fluorescence from 0 to -0.6 within 600 seconds, and reaching -0.8 within 800 seconds (Figure 5D). Interestingly, 20 μM sphingosine did not increase intracellular free calcium, measured by Fluo4 and in the absence of external calcium (Figure 5D). Also, sphingosine dereased mitochondrial membrane potential (Figure 5D). However, the rate of decrease was significantly slower than that induced by FTY-720. Sphingosine decreased mitochondrial membrane potential from 0 to -0.6 in approximately 800 seconds (Figure 5D), and reached -0.8 at approximately 1000 seconds (Figure 5D). To test whether the ability of FTY-720 in mobilizing calcium from mitochondrial compartments as well as acting on mitochondrial membrane potential would extend to other NB cell lines with MYCN amplification, experiments were performed using IMR32, SK-N-Be1 and SK-N-Be(2)c. In all MYCN-amplified cell lines FTY-720 mobilized calcium and disrupted membrane potential is similar kinetics as observed for the MYCN2 NB cell line (Supplementary Figure 4A–4I). Further calcium raised the PCC value for calcium and mitochondrial signal significantly in IMR32 as well as SK-NBe(2)c but failed to significantly increase in SK-NBe1 cells. The results show that FTY-720 and sphingosine decreased mitochondrial membrane potential but at different rates and to different degrees in multiple NB cell lines with and without MYCN amplification.

Finally, the effects of FTY-720 on mitochondrial permeability transition was determined in SHEP-1 NB cells treated with 10 μM FTY-720 or left untreated for 16 hours. The cells were loaded with calcein-AM and MitoTracker Red, with cobalt chloride to quench cytosolic calcein-AM. The results show that there was a loss of mitochondrial calcein-AM signal in cells treated with FTY-720 compared to untreated cells (Figure 5E), an indication of opening of the mitochondrial permeability transition pore. The results show that FTY-720 increases intracellular free calcium levels, induces loss of plasma membrane integrity and mitochondrial membrane potential, and induces the opening of the mitochondrial permeability transition pore.

### FTY-720 alters intracellular calcium signaling

**Figure 6:**
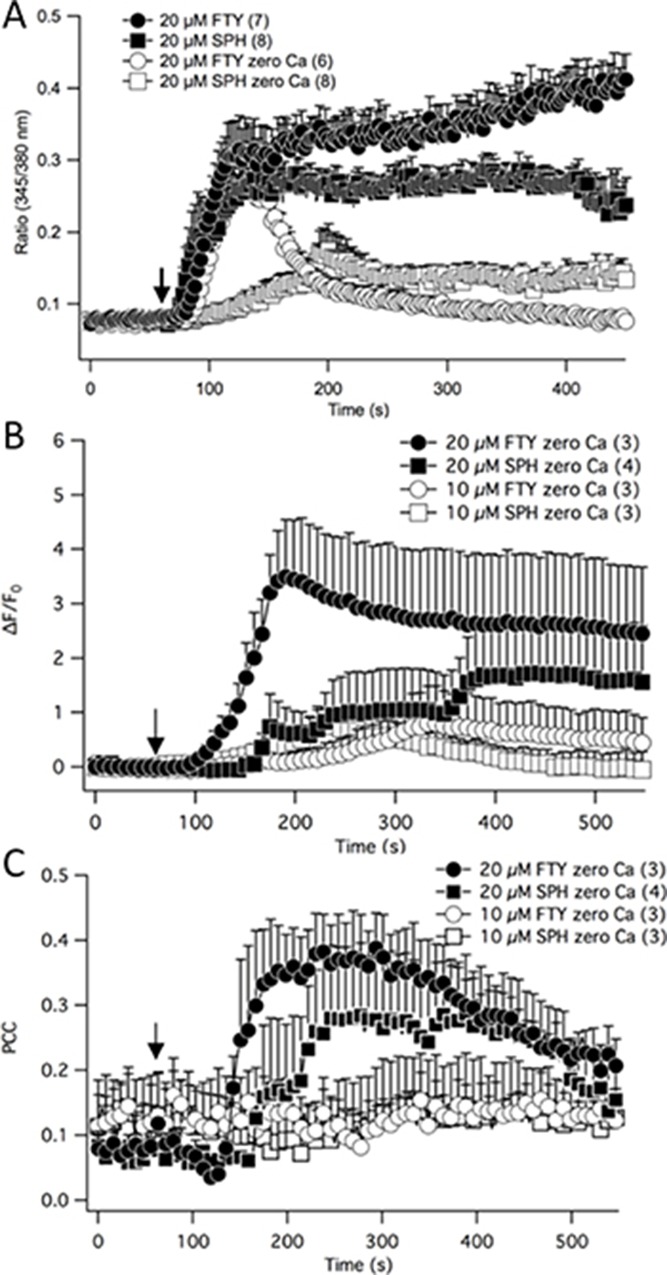
Fura-2 ratiometric calcium measurements were recorded in neuroblastoma cells treated with FTY-720 or sphingosine in the presence or absence of external calcium (**A**). Intracellular free calcium was measured through time-lapse confocal recordings in neuroblastoma cells by staining cells with Rhodamine 2 and MitoTracker (**B**). The Pearson's Correlation Coefficient was calculated to determine the mitochondrial calcium levels. All experiments were performed *n* = 3.

TRPM7 is a calcium permeable channel and calcium is a second messenger that regulates a plethora of fundamental physiological processes [22, 23]. Therefore, the effects of FTY-720 on NB calcium signaling were determined. NB cells were loaded with Fura-2 AM and ratiometric calcium measurements were obtained using a Zeiss microscope, monochromatic light source, and a photomultiplier. The results show that in the presence of external calcium and upon external application of 20 μM FTY-720, the intracellular free calcium rapidly increased and continued to steadily increase (Figure 6A). FTY-720 is an analogue of sphingosine. Therefore, in order to determine if the effects of FTY-720 on calcium signaling was due to sphingosine signaling, the experiment was repeated using sphingosine. In the presence of external calcium 20 μM sphingosine induced a rapid increase in intracellular calcium, reaching a peak of approximately 0.275 (Ratio 345/380 nm) (Figure 6A). However, the steady increase in intracellular free calcium observed with FTY-720 was not observed with sphingosine (Figure 6A). Next, in order to determine the effects of FTY-720 and sphingosine on intracellular calcium signaling, these experiments were performed in the absence of external calcium. In the absence of external calcium, 20 μM FTY-720 induced a rapid increase in intracellular calcium levels with the kinetics and peak fluorescence (Ratio 345/38 nm) that were comparable to the rapid increase observed in the presence of external calcium (Figure 6A). However, the intracellular free calcium levels dissipated quickly, reverting back to basal levels (Figure 6A). In the absence of external calcium, 20 μM sphingosine failed to induce the rapid increase in intracellular free calcium observed in the presence of external calcium, and the peak levels only reached ∼50% that observed in the presence of external calcium (Figure 6A).

Next, the effects of FTY-720 on mitochondrial calcium levels were examined. SHEP-1 NB cells were loaded with MitoTracker and Rhod-2, a mitochondrial specific calcium indicator dye. Time lapsed images of the cells were obtained using a Leica laser scanning confocal microscope to visualize changes in Rhod-2 levels in the mitochondria. Upon external application of 20 μM FTY-720, and with 0 external calcium, there was a significant increase in global Rhod-2 levels, indicating an increase in intracellular free calcium (Figure 6B). The increase in intracellular free calcium induced by 10 μM sphingosine, 20 μM sphingosine and 10 μM FTY-720 occurred at a much slower rate and only a fraction of the signaling induced by 20 μM FTY-720 (Figure 6B). The mitochondrial calcium levels were obtained by quantifying the co-localization of Rhod-2 signal and MitoTracker, and using the Pearson's Correlation Coefficient (PCC) to statistically measure the linear interdependence between the two signals. The results show that 20 μM FTY-720 increased the PCC approximately 4 fold from +0.1 to +0.4, suggesting that the treatment increased mitochondrial calcium levels (Figure 6C). To determine whether these effects were due to sphingosine signaling, sphingosine was used as a control. Similar results were obtained using 20 μM sphingosine, albeit to a lesser degree (Figure 6C), increasing the PCC from +0.1 to +0.3. In addition, 10 μM FTY-720 and 10 μM sphingosine did not have an effect on mitochondrial calcium levels (Figure 6C). The results show that there is a significant difference in the intracellular calcium signaling induced by FTY-720, compared to sphingosine. To test whether FTY-720's effect is of significance to a broader panel of NB cell lines with MYCN-amplified status, a series of experiments using IMR32, SK-N-Be1 and SK-N-Be(2)c were performed. In all cell lines FTY-720 mobilized mitochondrial calcium as indicated by PCC (Supplementary Figure 4D–4I).

### TRPM7 expression in NB patients

**Figure 7:**
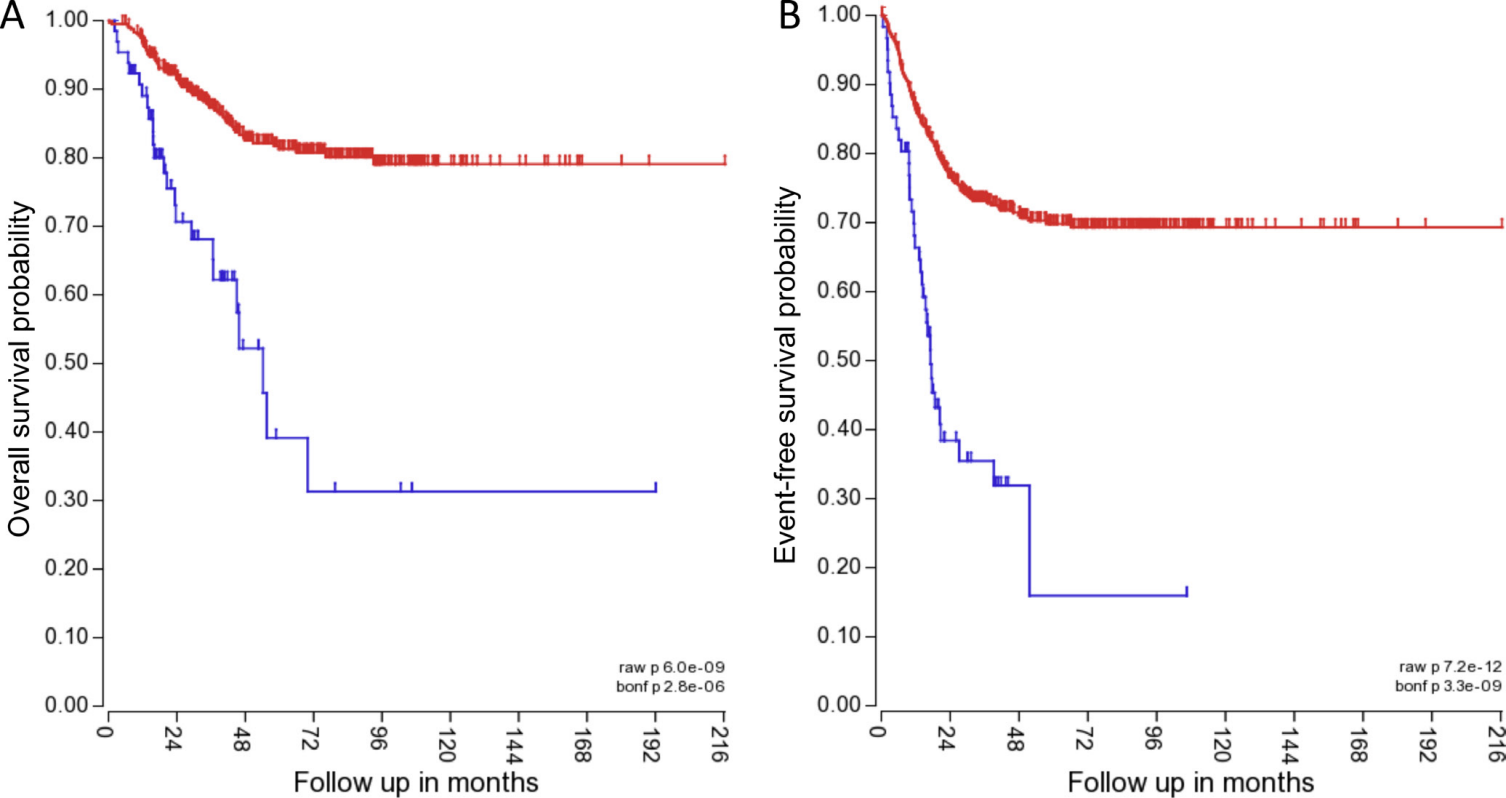
TRPM7 expression correlates with lower overall and event-free patient survival The blue line indicates high TRPM7 expression in 411 of 649 NB patient samples for overall patient survival, and 415 of 649 NB patient samples for event-free patient survival. The red line indicates high TRPM7 expression in 65 of 649 NB patient samples for overall patient survival, and 61 of 649 NB patient samples for event-free patient survival. The *P* value for overall survival probability is 6.0 × e^-09^, and for event-free survival probability it is 7.2 × e^-12^.

To determine the clinical relevance of TRPM7, the correlation of TRPM7 expression with NB patient outcome was determined. The Kocak tumor set and the R2 microarray analysis visualization platform was used (http://r2.amc.nl) to analyze TRPM7 expression in tumors from NB patients. The mRNA expression for TRPM7 was analyzed using Affymetrix expression profiling. TRPM7 expression inversely correlated with higher overall survival probability and event-free survival probability. 411 patients with low TRPM7 expression had an overall survival probability of ∼80% and event free survival probability of ∼70% (Figure 7A and 7B). 65 patients with high TRPM7 expression had an overall survival probability of ∼30% and event-free survival probability of ∼15% (Figure 7A and 7B). The data suggests that TRPM7 expression correlates with lower patient survival.

## DISCUSSION

Neuroblastoma is the most common extra-cranial solid pediatric tumor. Relapsed high-risk NB (∼40–50% of patients) signifies poor prognosis, and is difficult to treat due to the lack of response to current therapies and aggressive disease progression. Novel drugs and alternative treatments are being investigated for patients with relapsed high-risk NB. However, finding an effective treatment strategy for these patients continues to be a major challenge due to aggressive phenotypes of these tumors and a myriad of complex mechanisms that promote resistance to treatments and recurrence [1, 2, 4–6]. The current study focuses on examining the effects of FTY-720 or fingolimod an FDA-approved drug for refractory multiple sclerosis, in NB [10, 11]. The current study revealed that FTY-720 has multiple effects on NB cells that ultimately reduces NB cell viability and induces NB cell death in a time- and dose-dependent manner. The observed effect extends to different cell lines tested of different MYCN-status however the efficiency of FTY-720 in inducing cell death seems to correlate with TRPM7 expression levels. Overexpression of MYCN, which has been reported to lead to increased levels of TRPM7 [24, 25], slightly shifts the IC_50_ value of FTY-720 to lower levels in the SHEP NB cells. FTY-720 effects were similar in IMR32 NB cells, which express high functional TRPM7 compared to MYCN non-amplified cell lines cells lines as previously reported [25]. Strikingly, when comparing two cell lines derived from the same patient at the time of diagnosis where patient was responsive to treatment (SK-N-Be1) and after relapse and development of multi-drug resistance (SK-N-Be(2)c), the apoptosis inducing effect of FTY-720 was more potent in the drug-resistant cell line compared to the drug sensitive cell line. The drug resistant cell line exhibited higher TPRM7 activity, as shown in whole-cell patch clamp recordings (unpublished data), and expressed higher levels of TRPM7 as indicated by western blot analysis, when compared to drug sensitive cell line. Further FTY-720 induced immediate loss of membrane potential in all cell lines tested however it was difficult to detect PCC increase for mitochondrial calcium only in SK-N-Be1. This may be due to decreased cell size of drug sensitive NB cells, compared to drug resistant NB cells. Capacitance measurements are an indicator for cell size and were at an average of ∼3 pA/pF versus ∼ 6 pA/pF for SK-N-Be1 and Sk-N-Be(2)c respectively (values obtained from 12–15 measurements (data not shown). This observation suggest that the effect of FTY-720 (i.e. induction of cell death) may be regulated through multiply signaling pathways, one being a direct action on TRPM7 itself.

Interestingly, western blot analysis of whole cell lysates showed that 3 μM FTY-720 induced autophagy, as shown by the increase in LC-3 II. However, it had no effect on PARP cleavage, an apoptosis marker. Higher concentrations of 10 μM FTY-720 showed an increase in LC-2 II and PARP cleavage. This suggests that FTY-720 may induce autophagy when administered in low concentrations (e.g. 3 μM FTY-720) as a compensatory response to promote NB cell survival. However, at high concentrations (e.g. 10 μM FTY-720), the mitochondrial calcium signaling and loss of mitochondrial membrane potential results in damage to mitochondria that may be too extensive and the autophagic response may no longer be sufficient to promote NB cell survival, thereby resulting in NB cell death [14, 15].

The mRNA expression of NB patient tumor samples (Kocak tumor set) was analyzed for TRPM7 using Affymetrix expression profiling. TRPM7 expression correlated with higher overall survival probability and event-free survival probability. The data suggests that TRPM7 expression correlates with lower overall and event-free patient survival. Therefore, the effects of FTY-720 on TRPM7 were examined. FTY-720 is a modulator of sphingosine-1-phosphate receptor [10], and other groups have found that FTY-720 and sphingosine inhibits TRPM7 in HEK293 cells over-expressing TRPM7 and in cardiomyocytes [13]. Therefore, the effect of FTY-720 and sphingosine on TRPM7 was examined in NB cells. The results showed that FTY-720 rapidly inhibits TRPM7 channel activity, and cessation of drug application restores TRPM7 currents. FTY-720 also inhibited TRPM7 kinase activity, as shown by a decrease in the phosphorylation of TRPM7 kinase targets myosin IIA heavy chain and histone H3. The targeting of myosin IIA and histone H3 by TRPM7 kinase in NB cells [26–30] was confirmed by down-regulating TRPM7 expression with TRPM7 specific siRNA. Transfection of NB cells with TRPM7 siRNA significantly decreased myosin IIA and histone H3 phosphorylation compared to cells transfected with scrambled control siRNA and mock transfected cells (lipofectamine only). In addition, sphingosine had no effect on TRPM7 channel (Figure 3) and kinase activities (data not shown). Therefore, FTY-720 may exert its anti-cancer effects through multiple pathways, and one of the pathways directly involves TPRM7 and is independent of sphingosine signaling. Interestingly, higher concentrations of FTY-720 (20 μM) inhibited TRPM7 currents but also activated another TRP-like channel, which has a current-voltage relationship that is similar to non-selective TRP channels, linear current voltage relationship and crossing the x-axis near 0mV. Therefore, in addition to its effects on TRPM7, FTY-720 may also have other effects that account for its anticancer effects in NB.

Due to the fact that TRPM7 is a calcium-permeable ion channel [22, 31, 32], and that low concentrations of FTY-720 inhibits TRPM7 but higher concentrations activates another TRP-like channel, experiments were performed to investigate the calcium signaling in NB cells treated with FTY-720, sphingosine and in control untreated cells. The results showed that FTY-720 modulates calcium signaling, intracellular free calcium levels and mitochondrial calcium, in a dose-dependent and time-dependent manner. At 3 μM FTY-720, no changes in calcium levels were observed. At 10 and 20 μM FTY-720, the intracellular free calcium levels increased within 1 hour, when fluorescence was measured using the Perkin Elmer Operetta imaging system. However, when the fluorescence intensity was measured using a laser scanning confocal microscope, 20 μM induced a rapid increase in intracellular free calcium levels, compared to control. Further analysis of mitochondrial calcium levels showed that 20 μM FTY-720 but not 10 μM FTY-720 induced a rapid increase in mitochondrial calcium levels, as shown by the Pearson's Correlation Coefficient analysis of Rhod-2 and MitoTracker co-localization. These effects were not observed in cells treated with 10 μM Sphingosine, and 20 μM Sphingosine induced changes in intracellular free calcium levels and mitochondrial calcium levels but to a lesser degree. Interestingly, as electrophysiological measurements showed, 20 μM FTY-720 inhibited TRPM7 currents but also induced a TRP-like channel. The TRP-like channel that was induced by 20 μM FTY-720 may play a role in the increase in intracellular free calcium levels and mitochondrial calcium levels. This effect could also be due, in part, by sphingosine signaling. Interestingly, 20 μM FTY-720 induced a loss of mitochondrial membrane potential, and the opening of the mitochondrial permeability transition pore. These results suggest that FTY-720 may cause mitochondrial damage [33, 34], and at low concentrations of FTY-720, NB cells may induce autophagy of damaged mitochondria to promote NB cell survival [14, 15, 33]. However, at higher concentrations of FTY-720, the damage to mitochondria may be extensive and autophagy may not be sufficient to maintain NB cell viability. Interestingly, the data also showed that low concentrations of FTY-720 sensitized drug-resistant NB cells to doxorubicin, and other antineoplastic drugs (data not shown). This effect makes FTY-720 an attractive alternative for the treatment of NB patients, as it is effective on multiple NB cells, including the drug-resistant cell line SK-N-Be(2)c [35].

The current study revealed multiple pathways that are altered by FTY-720, which ultimately leads to NB cell death. Interestingly, other studies on the effects of FTY-720 in NB showed that FTY-720 induces NB cell death by inhibiting sphingosine kinase 2 and interfering with the sphingolipid pathway [12], and enhances the anticancer effects of topotecan. They were able to show the efficacy of FTY-720 on NB tumor growth *in vivo* using a NB xenograft mouse model. The current study also showed that there is a sphingosine effect in NB cells *in vitro*. However, there are multiple targets of FTY-720, and all of these effects play a role in inducing NB cell death.

In conclusion, FTY-720 induces mitochondrial calcium signaling, loss of mitochondrial membrane potential, opening of the mPTP and apoptosis. Sphingosine signaling, inhibition of TRPM7 channel and/or kinase activities, and activation of a TRP-like channel may play a role in these processes.

## MATERIALS AND METHODS

Cell culture MYCN2 cells, a tetracycline inducible MycN over-expression NB cell line (provided by Jason Shohet) as well as IMR32, SK-N-Be1 and SK-N-Be(2)c were authenticated by the cell line authentication testing services at Genetica DNA laboratories (USA) using STR DNA typing to verify each cell line and purity against contamination. All experiments were performed at less than 20 cell passages. The cells were maintained in RPMI-1640 (Mediatech, Inc., Manassas, VA, USA) containing 10% (v/v) heat-inactivated fetal bovine serum (FBS) (Atlanta Biologicals, Lawrenceville, GA, USA), and grown at 37°C, 5% CO_2_, in a 95% humidity.

### Affymetrix DNA micro-array hybridization and analysis

There are expression profiles for 649 NB tumors in the Kocak NB tumor dataset. This tumor set has documented genetic and clinical features. Briefly, total RNA was extracted from frozen NB tumors, and labeled cRNA was used to determine single-color gene expression profiles using GeneChip operating software (MAS5.0 and GCOS1.0, from Affymetrix), as previously described [16]. The NB tumor sets were accessed through the Gene Expression Omnibus (GEO) database at the NCBI website (GSE45547). The Kocak NB tumor set was used to analyze TRPM7 expression with overall patient survival and event-free patient survival. All analyses were performed using R2, an Affymetrix analysis and visualization platform developed in the Department of Oncogenomics at the Academic Medical Center at the University of Amsterdam. The “R2: Genomics Analysis and Visualization Platform” can be accessed at: http://r2.amc.nl. Statistical analysis was performed using SPSS version 20.0 (IBM, Mainz, Germany). The two-tailed Mann-Whitney *U*-test and Kruskal-Wallis tests, and unpaired two-tailed student's *t*-tests were used as appropriate. Kaplan-Meier estimates for overall survival and event-free survival were compared by log-rank test.

### Chemicals

General chemicals were from VWR (West Chester, PA). Doxorubicin and ionomycin were from Calbiochem (Gibbstown, NJ).

### Calcium assay

MYCN2 cells were washed and incubated with 1 μM Fluo-4 AM, the acetoxymethyl ester form of Fluo-4 (Molecular Probes, Eugene, OR, USA), for 30 minutes at 37°C in a standard modified Ringer's solution of the following composition (in mM): NaCl 145, KCl 2.8, CsCl 10, CaCl_2_ 2 (or 0), MgCl_2_ 2, glucose 10, Hepes·NaOH 10, pH 7.4, 330 mOsm. For nominally calcium free experiments 1mM EGTA was added to the external solution and calcium chloride was omitted. Cells were transferred to 96-well plates at 10,000 cells/well and stimulated as indicated. Epifluorescent measurements were performed using an Operetta High Content Imaging System (PerkinElmer, Santa Clara, CA, USA). Fluorescence intensity was quantified using Harmony (PerkinElmer, Santa Clara, CA, USA).

### Fluorescence measurements

MYCN2 cells were incubated in a standard modified Ringer's solution of the following composition (in mM): NaCl 145, KCl 2.8, CsCl 10, CaCl_2_ 2 (or 0), MgCl_2_ 2, glucose 10, Hepes·NaOH 10, pH 7.4, 330 mOsm. For nominally calcium-free experiments, 1 mM EGTA was added to the external solution and calcium chloride was omitted. Cells were loaded with fura-2 AM, the acetoxymethyl ester form of fura-2 Molecular Probes, Eugene, OR, USA. Cells were perfused with external solutions containing FTY-720, Spingosine, and cytosolic calcium was measured in individual cells using a Zeiss microscope and monochromatic light source tuned to excite fura-2 fluorescence at 360 and 390 nm for 20 msec each. Emission was detected at 450–550 nm using a photomultiplier.

### Time lapsed, live-cell imaging and pearson's correlation coefficient analysis

MYCN2 cells were plated on poly-D-lysine-coated coverslips. Cells were incubated and maintained in Ringer's extracellular media (in mM: NaCl 140, KCl 2.8, Na-HEPES 10 pH 7.2, CaCl_2_ 1, MgCl_2_ 1, Glucose 11, adjusted to 290 mOsm). To detect ER and calcium levels, cells were incubated with 2 μM Rhod-2-AM, 1 μM ER-Tracker Green and 1 mM Probenecid for 30 min at room temperature (RT). To detect mitochondria and calcium signals, cells were incubated with 2μM Rhod-2-AM, 0.5 μM of MitoTracker Green and 1 mM Probenecid for 30 min at RT. To detect calcium levels and mitochondrial membrane potential (Δ_Ψm_), cells were loaded with 2μM Fluo-4-AM and 1mM Probenecid for 45 min at RT, and then 20 nM tetramethylrhodamine ethyl ester (TMRE) was added for additional 15 min at RT. Single-cell time-lapse recordings were acquired using a Leica DMI 400B confocal microscope using a Leica 63X/1.40 oil immersion objective, and appropriate excitation lasers and emission filters. Images were obtained every 3.96 sec. A micro perfusion system was used (Picospritzer III, Parker) to apply different drug treatments using glass borosilicate micropipettes pulled in a horizontal puller (DMZ-Universal puller, Zeitz), driven by micromanipulator (MPC-200, Sutter Instruments).

The time-lapse images were processed on Volocity software (Perkin-Elmer). The calcium levels were calculated and informed as changes in relative fluorescence over time as published elsewhere [17]. Briefly, for each timepoint six regions of interest (ROI) were selected on the cell soma (F_raw_) where signals from both channels were present (to ensure further colocalization calculations), and their mean intensity were averaged. Three ROIs were selected randomly on the background (F_bg_) and averaged. The average background signal was subtracted from the averaged raw to produce background-corrected ROIs (F_ROI_, see Equation 1).

$$F_{\text{ROI}}=F_{\text{raw}}-F_{b}$$ (1)

The basal fluorescence of the cells (F_0_) was calculated by averaging the F_ROI_ values of the cells in their resting state, before the perfusion of the drug. Finally, relative changes of fluorescence over time was calculated as depicted in Equation 2:

$$\frac{DF}{F_{o}}=\frac{\left(F_{\text{ROI}}-F_{o}\right)}{F_{o}}$$ (2)

For the colocalization, a Pearson's Correlation Coefficient (PCC) was calculated for each ROI using the threshold method [18] in the Volocity software and averaged on each time point. Further calculations were carried out on a Python 3.2 script and data was plotted on Igor Pro 6 (Wavemetrics).

### Mitochondrial permeability pore transition

NB cells were treated with FTY-720 or left or vehicle. After treatment, the cells were stained with 1 μM Calcein AM and 0.2 μM MitoTracker Red in Hanks’ Balanced Salt Solution (HBSS) with sodium bicarbonate, calcium, and magnesium that also included HEPES (10 mM), L-glutamine (2 mM) at 37°C for 15 minutes. The cells were washed in buffer, and images were acquired using a Leica DMI 400B laser scanning confocal microscope.

### Cell viability and proliferation assay

The Sulforhodamine B (SRB) colorimetric assay was used to determine cell proliferation following the protocol previous described [19]. Briefly, cells were seeded at a density of 10,000 cells/ well on a transparent, flat-bottom, 96-well plate and allowed to settle overnight. At the initiation of each experiment (*t* = 0), and after drug treatments, 100 μL of 10% (w/v) Trichloroacetic acid were added to each well, incubated for 1 h at 4°C, washed with deionized water, and dried at room temperature. 100 μl of 0.057% (w/v) SRB solution were added to each well, incubated for 30 min at room temperature, rinsed four times with 1% (v/v) acetic acid, and allowed to dry at room temperature. Finally, 200 μL of 10 mM Tris base solution (pH 10.5) were added to each well, and after shaking for 5 min at room temperature, the absorbance was measured at 510 nm in a microplate reader. The absorbance at *t* = 0 was compared with the absorbance at the end of the experiment to determine cell growth in treated cells compared with control cells. The CellTiter 96^®^ AQ_ueous_ One Solution Promega was performed as previously described [20].

### Western blot analysis

Cell lysates were prepared in radioimmunoprecipitation assay buffer [20 mmol/L Tris-HCl (pH 7.5), 0.1% (w/v) sodium lauryl sulfate, 0.5% (w/v) sodium deoxycholate, 135 mmol/L NaCl, 1% (v/v) Triton X-100, 10% (v/v) glycerol, 2 mmol/L EDTA] supplemented with a protease inhibitor cocktail (Calbiochem, EMD Millipore Corporation, MA, USA) and phosphatase inhibitors sodium fluoride (20 mmol/L) and sodium vanadate (0.27 mmol/L). Western blot analysis was done as previously described [21]. The total protein concentration was determined using the protein assay dye reagent from Bio-Rad Laboratories. Cell lysates in SDS-sample buffer were boiled for 5 min and equal amounts of total protein were analyzed by 10% SDS-PAGE and Western blotting. The antibodies used in this study were obtained from Cell Signaling Technology (Danvers, MA, USA). Antibodies detecting full length and cleaved TRPM7 were obtained from Abcam (Cambridge, UK) and Santa Cruz Biotechnology (Dallas, Texas)respectively. Proteins were detected using the Odyssey Infrared Imaging System (LI-COR Biosciences, Lincoln, NB, USA) and analyzed with LI-COR Image Studio 2.0 acquisition and analysis software. The density of each band was quantified using ImageJ analysis software.

### Statistical analysis

Results are shown as the mean + standard deviation. Statistical significance was determined based on a two-way analysis of variance (Student's *t*-test). Adjacent to data points in the respective graphs, significant differences were recorded as follows: single asterisk, *p* < 0.05. SRB and Fluo-4 experiments are all *n* of at least 3, in triplicates. All other experiments are all *n* of at least 3.

## SUPPLEMENTARY MATERIALS FIGURES


