# Supplementary material for: FTY-720 induces apoptosis in neuroblastoma via multiple signaling pathways
Source: Oncotarget. 2017 Nov 6;8(66):109985–99. doi: 10.18632/oncotarget.22452 (PMC5746359; doi:10.18632/oncotarget.22452)
Supplement: Supplementary file 1 [file oncotarget-08-109985-s001.pdf]

FTY-720 induces apoptosis in neuroblastoma via multiple signaling pathways

SUPPLEMENTARY MATERIALS

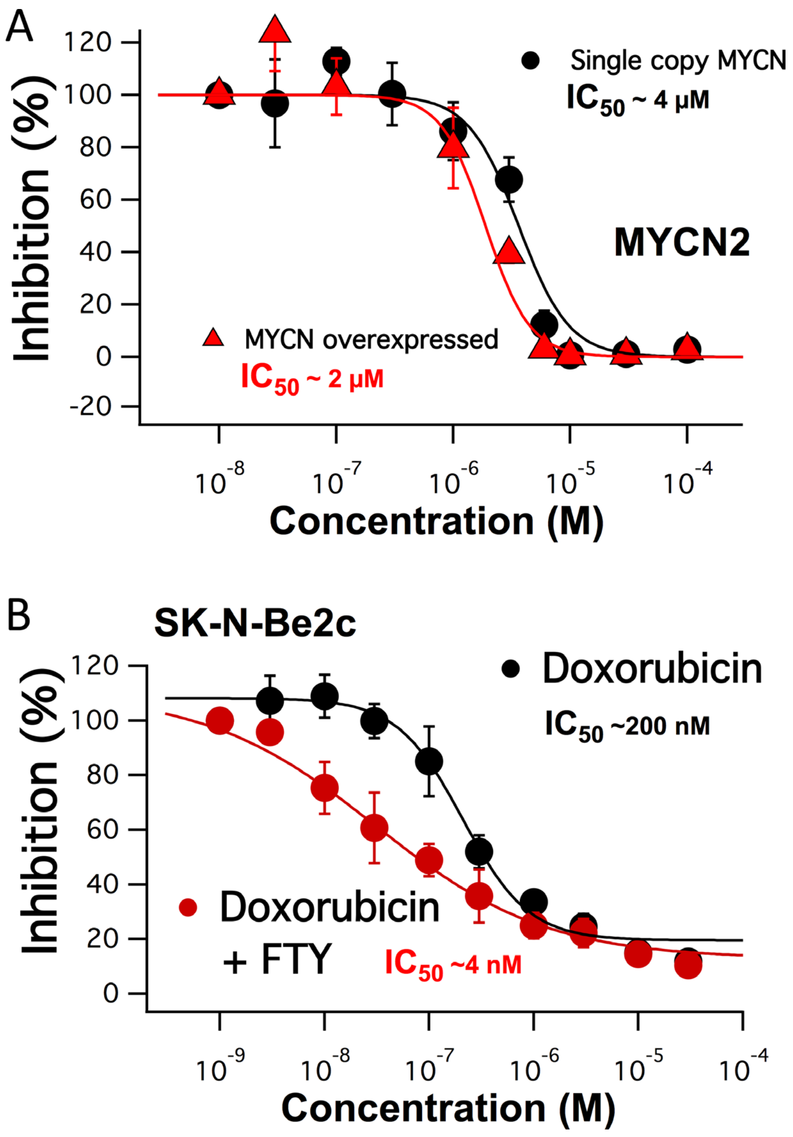

Supplementary Figure 1: (**A**) shows the dose dependent effects of doxorubicin in MYCN2 cells with and without induction of overexpression of MYCN2 obtained from SRB assays. (**B**) shows dose-dependent effect with and without FTY-720 pretreatment in SK-N-Be(2)c cells. All experiments were performed $n = 3$.

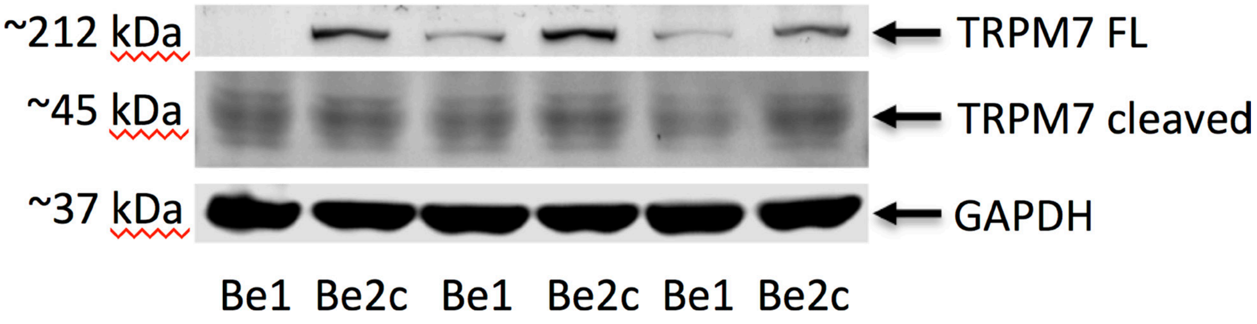

Supplementary Figure 2: Western blot analysis of whole cell lysates comparing TRPM7 expression from Neuroblastoma cell lines derived from the same patient before (Be1) and after development of multi-drug resistance (Be2c). All experiments were performed $n = 3$.

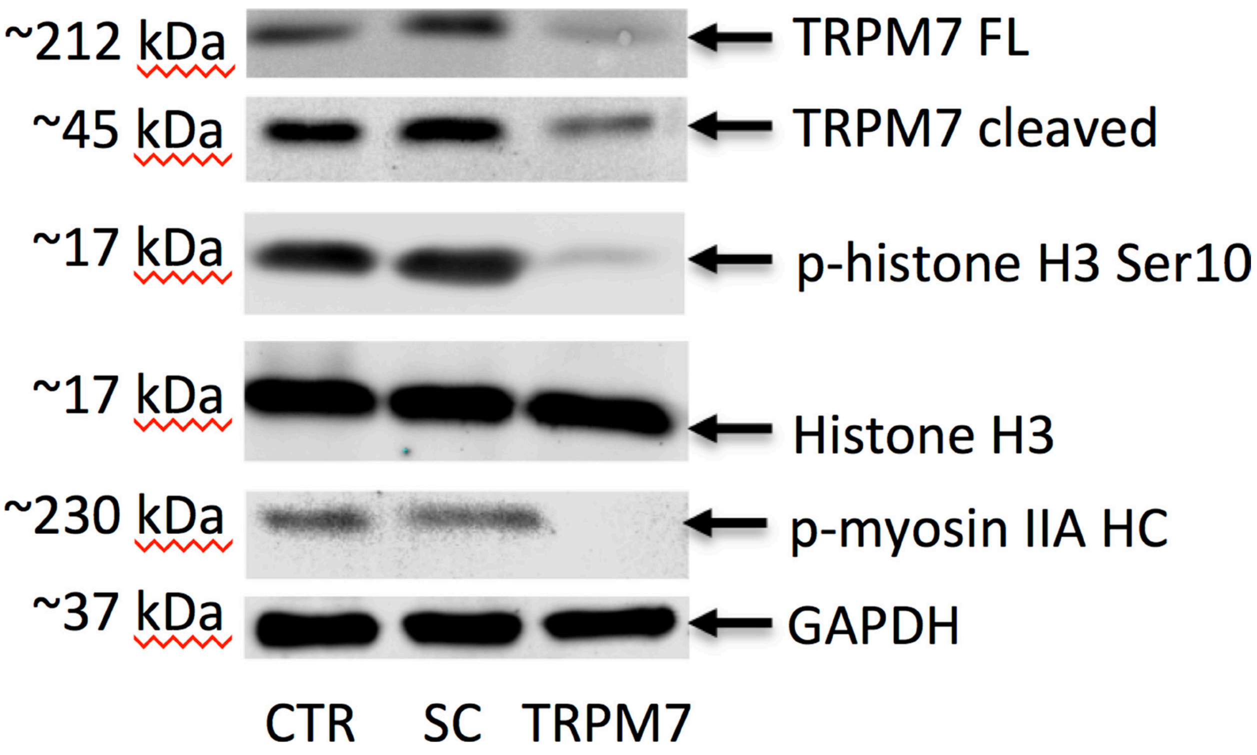

Supplementary Figure 3: Western blot analysis of whole cell lysates prepared from Neuroblastoma cells transfected with TRPM7 specific siRNA, scrambled siRNA or mock transfected. All experiments were performed. All experiments were performed $n = 3$.

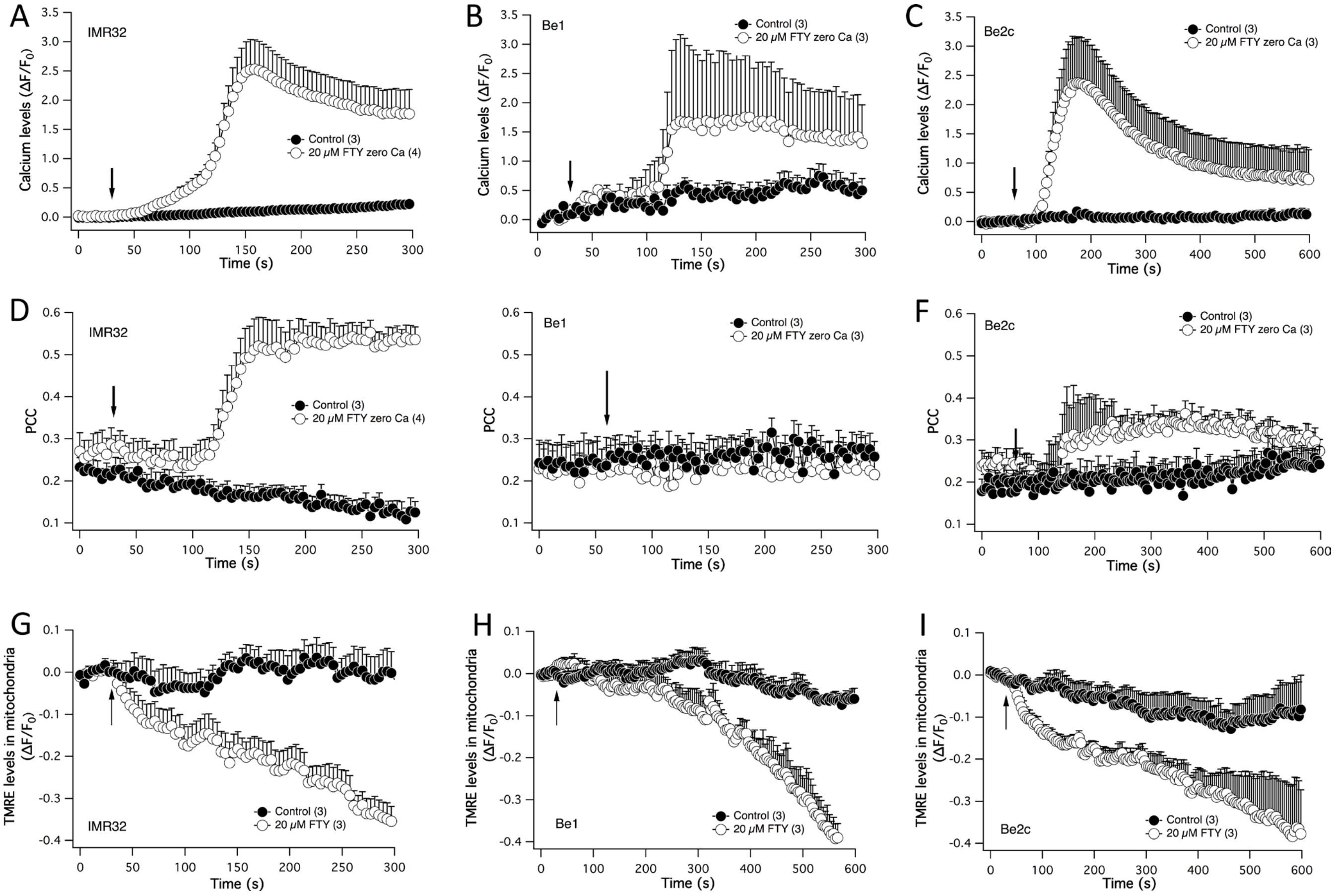

Supplementary Figure 4: Time-lapse confocal experiments were performed in IMR32, SK-N-Be1 and SK-N-Be(2)c cells to measure intracellular calcium levels (**A–C**), change in PCC of calcium and mitochondrial signal (**D–F**) and loss of mitochondrial membrane potential in response to application of 20 μM FTY-720 compared to control (**G–I**). All experiments were performed *n* = 3.
